# Protocol for sparse labeling of murine Purkinje cells by intracerebroventricular AAV injection using a microelectrode holder

**DOI:** 10.1016/j.xpro.2026.104719

**Published:** 2026-07-21

**Authors:** Jordan J. Lillibridge, Samantha Vancs, Martin M. Riccomagno, Fedor V. Karginov

**Affiliations:** 1 Molecular, Cell, and Systems Biology Department, University of California, Riverside, Riverside, CA 92521, USA

**Keywords:** Developmental biology, Microscopy, Gene Expression, Neuroscience

## Abstract

Most microinjection techniques require expensive machinery and can be difficult to perform consistently. Here, we present a protocol for long-term, stable gene delivery by adeno-associated virus (AAV) vectors through intracerebroventricular injection of neonatal mice using a microelectrode holder equipped with borosilicate needles. We describe steps for building the device, pulling the needles, preparing and injecting the neonates, tissue perfusion and collection, and sample preparation. We then detail procedures for immunofluorescent antibody staining and imaging, followed by image reconstruction, export, and analysis.

## Highlights

• Steps for building a microinjection device using a microelectrode holder
• Procedures for sparse labeling of Purkinje cells suitable for single-cell analysis
• Instructions for mouse perfusion and tissue sample preparation for immunofluorescence

## Abstract (editor highlights)

Publisher’s note: Undertaking any experimental protocol requires adherence to local institutional guidelines for laboratory safety and ethics.

## Graphical abstract

**Figure:**
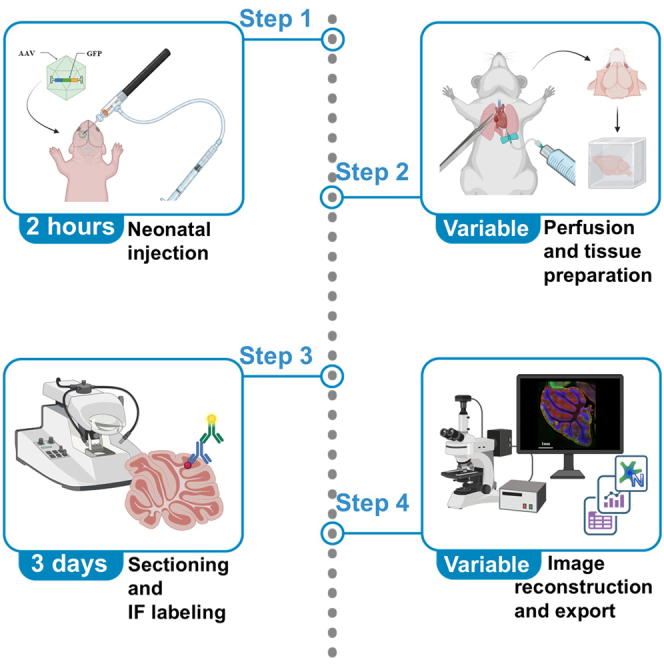

## Teaser

Most microinjection techniques require expensive machinery and can be difficult to perform consistently. Here, we present a protocol for long-term, stable gene delivery by AAV vectors through intracerebroventricular injection of neonatal mice using a microelectrode holder equipped with borosilicate needles. We describe steps for building the device, pulling the needles, preparing and injecting the neonates, tissue perfusion and collection, and sample preparation. We then detail procedures for immunofluorescent antibody staining and imaging, followed by image reconstruction, export, and analysis.

## Before you begin

We have devised a system for sparse labeling of Purkinje cells in neonatal mice using a microelectrode holder injection device and common laboratory reagents to deliver AAV8-GFP by intracerebroventricular (ICV) injection. Following microinjection, 10-weeks-old mouse brains were sectioned, confocal images of individual Purkinje cells were reconstructed in 3D using MBF Bioscience’s Neurolucida 360 software, and analyzed with Neurolucida Explorer. This protocol facilitates consistent sparse labeling of Purkinje cells for single-cell analysis, comparable to that of previous studies.^1^ Here, we find that the use of a microelectrode holder as the handle of the microinjection device performs comparably to previously described ICV methods, but can offer finer control by providing a stable grip for the investigator. We envision this as an effective adaptation to traditional microinjection techniques, increasing the accessibility, economic feasibility, and user control for neonatal ICV microinjections.

### Innovation

Over the last few decades, microinjection has revolutionized developmental biology and neuroscience. However, most microinjection techniques require expensive machinery and can be difficult to scale. Microinjection syringes can be used as stand-ins for stereotactic machinery, but consistently injecting sub-microliter quantities with a microsyringe is difficult to achieve, and replacement needles can be costly. Here, we describe a procedure for long-term, stable gene delivery by AAV vectors through intracerebroventricular injection of neonatal mice using a microelectrode holder equipped with borosilicate needles. Further, we adapt this approach to sparse labeling of Purkinje cells to allow for detailed morphological and other image analysis of single neurons. Taken together, this procedure recapitulates results seen from other injection methods while increasing accessibility, economic feasibility, and manual control of neonatal microinjection.

### Institutional permissions

All animal work was done with permission from the University of California, Riverside IACUC. It is important for readers to obtain permission from their institutional animal use committee before working with vertebrate animals.

## Key resources table

**Table:** 

REAGENT or RESOURCE	SOURCE	IDENTIFIER
**Antibodies**		

Chicken polyclonal anti-GFP (1:500)	Aves Labs	Cat# GFP-1020, RRID:AB_10000240
Rabbit anti-Calbindin (1:500)	Swant	Cat# CB38, RRID:AB_10000340
Goat anti-Chicken IgY (H+L) Secondary Antibody, Alexa Fluor™ 488 (1:1000)	Thermo Fisher Scientific	Cat# A-11039, RRID:AB_2534096
Goat anti-Rabbit IgG (H+L) Highly Cross-Adsorbed Secondary Antibody, Alexa Fluor™ 546 (1:1000)	Thermo Fisher Scientific	Cat# A-11035, RRID:AB_2534093

**Bacterial and virus strains**		

pAAV-CAG-GFP	The Edward Boyden Lab	RRID:Addgene_37825
AAV8-CAG-GFP	Addgene	Addgene viral prep # 37825-AAV8

**Chemicals, peptides, and recombinant proteins**		

Powdered PFA	Sigma-Aldrich	CAT#SIAL-158127-100G
DAPI	Invitrogen	CAT#62248
Ampule PFA	Thermo Scientific	CAT#28908
Agarose I	VWR Life Sciences	CAT#710
Fast green FCF	Sigma-Aldrich	CAT#F7252-5G
Clear or black nail polish	Any cosmetic supply store	N/A
Sodium azide	Sigma-Aldrich	CAT#S2002-5g
Goat serum	Sigma	CAT#G6767
Triton X-100	Fisher	CAT#AAA16046AE
Bovine serum albumin (BSA)	Sigma-Aldrich	CAT#A4737-25g
Fluoro Gel with DABCO	Electron Microscopy Sciences	CAT#17985–02

**Experimental models: Organisms/strains**		

Mouse (10 weeks old: male and female): C57BL/6JGpt	Gem Pharmatech	RRID:IMSR_GPT:N000013

**Recombinant DNA**		

pAAV-CAG-GFP	The Edward Boyden Lab	RRID:Addgene_37825
AAV8-CAG-GFP	Addgene	Addgene viral prep # 37825-AAV8

**Software and algorithms**		

Neurolucida 360	MBF Bioscience	N/A
Neurolucida Explorer	MBF Bioscience	N/A

**Other**		

Tygon∗ R-3603 Clear Laboratory Tubing, Diameter: 1/16 in., Wall Thickness: 1/32 in.	Fisher	CAT#14171129
Microelectrode Holder MP-S10A	Warner Instruments	CAT#64-1261
Borosilicate glass capillaries	World Precision Instruments	CAT#TW100F-4
Molding clay	Any arts and crafts store	N/A
10 cm Petri dishes	Falcon	CAT#351029
1 mL syringe (slip tip)	Vitality Medical	CAT#309659
Aramis Laboratory Animal Microtattoo System	Ketchum Manufacturing	CAT#623
Small animal heated pad	K&H	N/A
Parafilm	Heathrow Scientific Products	CAT#HS234526B
Surflo Winged Infusion Sets (Butterfly needles)	Terumo	CAT#22258092
6 well plates	CellPro	CAT#TPN1006-NT
Standard dissection kit	Carolina Biological	CAT#621290
50 mL syringe (Luer lock)	Fisher Scientific	CAT#13-689-8
Glass microscopy slides	Corning	CAT#2948
Cover slips	Fisher Scientific	CAT#12-544-DP
Confocal Microscope	Leica	CAT#17985–02
Needle pulling machine	Narishige	CAT#pc-10
Vibratome	Leica	CAT#VT1000S

## Materials and equipment

**Table:** Permeability solution

Reagent	Final concentration	Amount
10× PBS	1×	50 mL
Bovine serum albumin (BSA)	3%	15 g
Triton X-100	0.3%	1.5 mL
10% NaN_3_ (Sodium azide)	0.03%	1.5 mL
ddH_2_O	N/A	Fill to 500 mL
**Total**	**N/A**	**500 mL**

Store at 4C for up to 3 months.

**CRITICAL:** Sodium azide is very toxic when ingested and dangerous when in contact with skin. Be sure to wear gloves, goggles, and a lab coat when handling.

## Step-by-step method details

### Building the device and pulling needles

**Timing: 1 h**

This section describes how to use a microelectrode holder as the handle of the microinjector and how to pull borosilicate rods into needles. Both procedures can be done ahead of time. Needles are secured to a 10 cm Petri dish with a strip of modeling clay.

**Figure 1:**
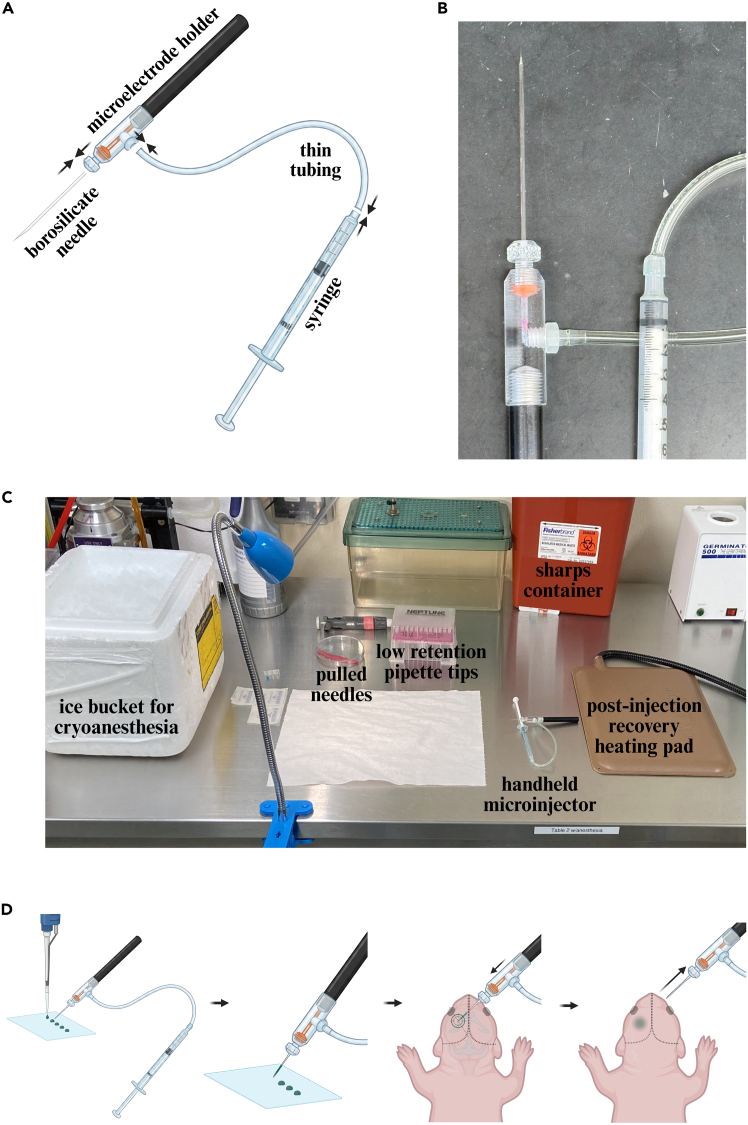
Construction of the microinjector and setup for neonatal ICV injections (A) Schematic reflecting assembly of the handheld microinjector from the recommended materials. (B) A zoomed in image shows the geometry of the needle and the connection points of the tygon tubing. (C) An example workstation set up for administration of neonatal cryoanesthesia, subsequent intracerebroventricular injection and post-injection recovery. (D) The FastGreen-AAV mixture is aliquoted in 1 μL droplets on parafilm using a P2 pipette. The pulled and beveled borosilicate capillary needle is brought to an aliquot of FastGreen viral mixture and capillary action facilitates loading of the target injection volume into the needle tip. The needle is positioned as shown, and the AAV is injected with very light force. The FastGreen should show as a diffuse circle around the site of injection.
2. Pull the borosilicate rods into needles:
  a. Parameters should be set such that the needle point is a centimeter in length.

***Note:*** On the Narishige PC-10 model, we used a 1 step program with the heater level set to 61.7.

1. Assemble the device according to Figure 1A.
  a. Secure the Tygon tubing to the port on the microelectrode holder.
    ***Note:*** Typically, 10–15 cm of Tygon tubing is sufficient, but length can be adjusted for comfort.
  b. Insert the 1 mL syringe slip tip into the open end of the Tygon tubing.
    ***Note:*** Close-up images of these connection points can be found in Figure 1B.

**CRITICAL:** The tip of the needle should be 0.5–1.5 cm in length with a diameter of approximately 60 micrometers. A representative image of the needle can be found in Figure 1B.

### Preparing the viral mixture for injection

**Timing: 30 min**

In this step, we thaw and dilute aliquots of AAV in preparation for injection. In our experience, 3.3 × 10^10^ Genome Copies (1.65 × 10^10^ GC per hemisphere) was sufficient to label approximately 20% of Purkinje cells. It is suggested that investigators use this as a starting point for optimization.

**CRITICAL:** For optimal outcomes, microinjections must be performed on P1 neonates and younger.

**CRITICAL:** Before your viral solution is prepared, count the number of pups you will be injecting and make enough for one extra pup. This limits waste and minimizes freeze-thaws.

3. Aliquot and freeze AAV in small volumes (1–5 μL).
4. When ready to use, thaw appropriate AAV aliquots on ice.

**CRITICAL:** Freeze thaws drastically reduce AAV efficiency. Thaw only enough virus for immediate use.

5. Dilute AAV to desired titer.
  a. Add FastGreen to a final concentration of 1× before you are ready to inject.

***Note:*** Keep on ice.

### Preparing and injecting the pups

**Timing: 1**–**3 h (depends on pup quantity and vivarium accessibility)**

This section describes a stepwise process to set up your injection area and perform the neonatal injections.

6. Gather the materials and arrange your workstation as in Figure 1C.
  a. Bring the small animal heating pad up to temperature and line with a paper towel.
7. Aliquot the FastGreen-AAV mixture in 1 μL droplets on parafilm using a P2 pipette.

***Note:*** See troubleshooting for guidance on this step.

**CRITICAL:** Prepare enough for 1–2 pups, this ensures minimal evaporation during the procedure. Avoid inaccurate injection volumes.

***Note:*** As speed and skill increases, more aliquots can be prepared.

8. Cryoanesthetize pups by gently surrounding them with crushed ice until their pink hue recedes, and they are nonresponsive to gentle paw pinches (∼2 mins).

***Note:*** Use a thin barrier, such as a glove, between the pups and the ice, as appropriate for your IACUC protocol

**CRITICAL:** Ensure that you are ready to complete the steps that follow quickly, so that the pups are not cryoanesthetized for too long.

9. Loosen the cap of the microinjector and insert the pulled needle. Carefully twist cap to tighten.
  **CRITICAL:** Overtightening will break the needle.
  a. Bevel the needle with a sharp pair of scissors at a 30 degree angle to the needle axis.
  b. Pull the plunger of the 1 mL syringe to 0.6–0.9 mL.
10. Bring the beveled tip to the 1 μL aliquot on the parafilm.

***Note:*** 1 μL of the viral mixture should be drawn into the needle tip by capillary action (See troubleshooting for guidance on this step).

11. Position the pup ventral side down facing towards the investigator.
  a. Insert the needle perpendicular to the center of the parietal plate, approximately 1 mm lateral to the sagittal suture and 1 mm posterior to the eye.
    ***Note:*** The triangulation of the injection site is shown in Figure 1D.
  b. An initial resistance, followed by a brief puncture, will be felt.
  c. Move the needle slightly out after the initial crushing puncture to position it in the ventricle.
    ***Note:*** The needle needs to be deep enough to puncture the skull but should not be inserted more than ∼2–3 mm deep.
12. Inject the fluid by applying very light force on the syringe.
  ***Note:*** The entire contents of the needle will be evacuated very rapidly.
  **CRITICAL:** Introduction of air into the ventricle often causes immediate death. See troubleshooting for guidance on this step.
  a. When no more viral mixture remains, carefully withdraw the borosilicate needle from the ventricle.
    ***Note:*** The FastGreen should spread throughout the ventricles and become visible as a diffuse circle around the site of injection (Figure 1D).
    ***Note:*** A new borosilicate needle should be used for each pup
    ***Note:*** Used needles are to be disposed of in an appropriate biohazard sharps container.
    ***Optional:*** Tattooing the base of the tail or the paws for identification is suggested if you are using more than one construct, or need to distinguish injected and uninjected mice.
13. Recover the pups by placing them on the heating pad lined with a paper towel.

***Note:*** Pups should regain their pink hue and resume normal movement within ∼5 min.

14. Place the pups back in their home cage.

***Note:*** The Mother will quickly gather the pups back into her nest.

**CRITICAL:** Cannibalization can occur at this stage. See troubleshooting for guidance on this step.

### Mouse tissue perfusion

**Timing: 30 min (per mouse)**

In this section, 10-weeks-old mice are perfused with PBS and paraformaldehyde (PFA). Successful perfusion flushes blood from the circulatory system and stiffens the tissue, making downstream applications more consistent.

15. Set up for Transcardial perfusion:
  a. Assemble a mouse perfusion area in a fume hood, for example, a styrofoam board with push pins propped up at an angle in a clean rectangular waste container

***Optional:*** Add cloth/cotton pad underneath to catch spills.

16. Prep the perfusion materials:
  a. Make a 4% PFA solution from powder (see key resources table).
    ***Note:*** Powdered 4% (w/v) PFA, pH 7.2 can be dissolved up to 30 days in advance.
    **CRITICAL:** Use an N-95 mask or respirator, and dissolve powdered PFA in a fume hood. Powdered PFA is toxic if inhaled. Store in the dark at 4°C.
  b. Fill two 50 mL luer lock syringes: one with 4% PFA & one with 1× PBS.
17. Attach and prime the butterfly needle with 1× PBS.
18. Euthanize the mouse in a CO_2_ chamber for ∼2–3 min.

***Note:*** The flow rate for CO_2_ euthanasia systems should be set to displace 40%–60% of the cage volume per minute.

19. Remove the mouse from the CO_2_ chamber and squeeze paw or tail with sufficient force to confirm euthanasia before proceeding.
20. Pin each limb to the styrofoam.

***Note:*** Optimal pin positioning is through the palms of the hands and pads of the feet.

21. Dissect away surrounding tissue to access the heart.
  a. Open the thoracic cavity by carefully cutting both sides of the ribcage.

**CRITICAL:** The heart should still be beating during the start of the perfusion. This keeps blood from clotting and helps push the PBS through the circulatory system.

22. Insert the butterfly needle into the left ventricle parallel to the ventricular septum to avoid puncture.
  a. Use tweezers or dissection scissors to rupture the right atrium (Figure 2A).

**Figure 2:**
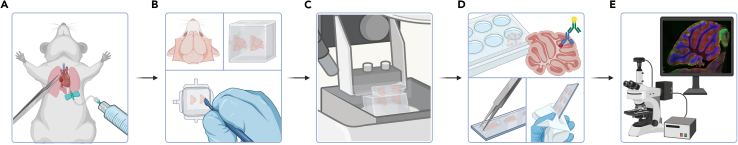
Workflow for tissue preparation, immunofluorescence, and imaging (A) 10-weeks-old mice are transcardially perfused with PBS followed by PFA. A butterfly needle is positioned in the left ventricle, followed by right atrium puncture, allowing for circulation of PBS, and subsequently PFA, through the circulatory system. (B) Following initial PFA fixing with transcardial perfusion, the brain is carefully dissected away from the surrounding tissue and, after additional incubation in fixing agent, is dried and agarose embedded for sectioning. (C) The agarose block is positioned according to the desired sectioning plane and mounted to a vibratome for sectioning. (D) Sections are incubated with antibodies for immunofluorescent staining, washed, and mounted on slides. (E) Sections are imaged using a confocal fluorescent microscope.
  b. Inject 1× PBS by administering constant gentle pressure on the 50 mL syringe.
    **CRITICAL:** If the butterfly needle was correctly placed into the left ventricle, the heart should visibly inflate, and the liver should begin to lighten in color.
  c. Continue until all 50 mL have been run through the circulatory system.
    ***Note:*** The PBS should start to run-off clear after ∼20 mL.
23. Hold the butterfly needle securely and switch butterfly needle tubing from the 1× PBS syringe to the 4% PFA syringe.
  a. Again, dispense the full 50 mL from the syringe with gentle constant pressure.

**CRITICAL:** Ensure that the needle does not shift position (see troubleshooting for guidance on this step).

***Note:*** The limbs, neck, and tail of the mouse should contract with the injection of 4% PFA.

**CRITICAL:** Complete injection of the 50 mL syringe is necessary to achieve successful perfusion (see troubleshooting for guidance on this step).

24. Remove the pins from the mouse.

***Note:*** If the perfusion was done correctly, the limbs should be very stiff.

### Brain tissue collection, post-fixation, and sectioning

**Timing: Variable**

In this step, whole brains are removed from the skull, post-fixed in paraformaldehyde, embedded in agarose, and sectioned using a vibratome.

25. Carefully dissect away the surrounding skin, muscle, and bone to extract the mouse brain tissue (Figure 2B).
  a. Incubate the brain tissue in 4% PFA for ∼4–16 h at 4°C in a 6 well plate (see Reference Table).

***Note:*** Dilute 16% PFA from ampules with 1× PBS to make the 4% PFA suitable for tissue fixation. This can be made up to 24 h in advance.

26. Remove the brain from the fixative and dry by gently rolling on a paper towel.
  a. Once the tissue is dry, place it into a mold and cover in a 3% Agarose/PBS solution (Figure 2B).

***Note:*** We used an empty coverslip box with approximate dimensions of 7 cm x 3 cm x 2 cm to block 3 brains at a time. Individual brains can be blocked in molds approximately 20 mm x 20 mm x 22 mm.

**CRITICAL:** Allow agarose solution to cool to just above the melting temperature before blocking, very hot agarose can harm the tissue.

27. Once solid, trim and mount the gel block to the vibratome, taking sectioning plane into account, and section tissue at a thickness of 50 μm (Figure 2C).

***Note:*** When processing multiple samples, agarose blocks can be cut to distinguish one sample from another when gathering the floating sections from the vibratome.

28. Sections can be collected into 6-well plates and stored in 1× PBS at 4°C.

***Note:*** Tissue regions of interest can be isolated before or after agarose blocking (e.g. the cerebellum and cerebrum can be separated before or after blocking).

Pause point: 1× PBS + 0.05% sodium azide (v/v) can be used for long term section storage. We observed sections remaining viable for more than 6 months when stored at 4°C in the dark.

### Immunofluorescent antibody staining and imaging

**Timing: 3 days**

Here we describe our immunostaining and imaging procedure.

29. Move the desired sections into a new 6-well plate.
30. Incubate with 1.5 mL of 1:500 Chicken polyclonal anti-GFP primary antibody + 5% goat serum + permeability solution at 4°C for 12–16 h with gentle rocking.
  ***Note:*** See materials for the permeability solution recipe.
  a. Wash sections with 1× PBS 5 min three times with gentle rocking.
    ***Note:*** 330 ng/mL DAPI may be added to any PBS wash as a nuclear counterstain.
31. Incubate with 1.5 mL of 1:1000 Goat anti-Chicken IgY (H+L) Secondary Antibody, Alexa Fluor™ 488 + 5% goat serum + permeability solution at 4°C for 12–16 h with gentle rocking.
  a. Wash sections with 1× PBS 5 min three times with gentle rocking.
32. Transfer each stained section to a glass slide and use a Kimwipe to dry any residual liquid by capillary action (Figure 2D).

**CRITICAL:** Do not touch the tissue directly with the Kimwipe as this can cause damage.

33. Dry for 10–20 min or until the tissue looks transparent.
34. Add mounting medium (See key resources table) and a coverslip.
  a. Cure for ∼20 min.
35. Seal the coverslip border with nail polish.

**Pause point:** Slides can be stored in the dark at 4°C.

***Note:*** We observed slides to be good for 3–6 months with thorough sealing. However, we recommend imaging them within a week.

**Figure 3:**
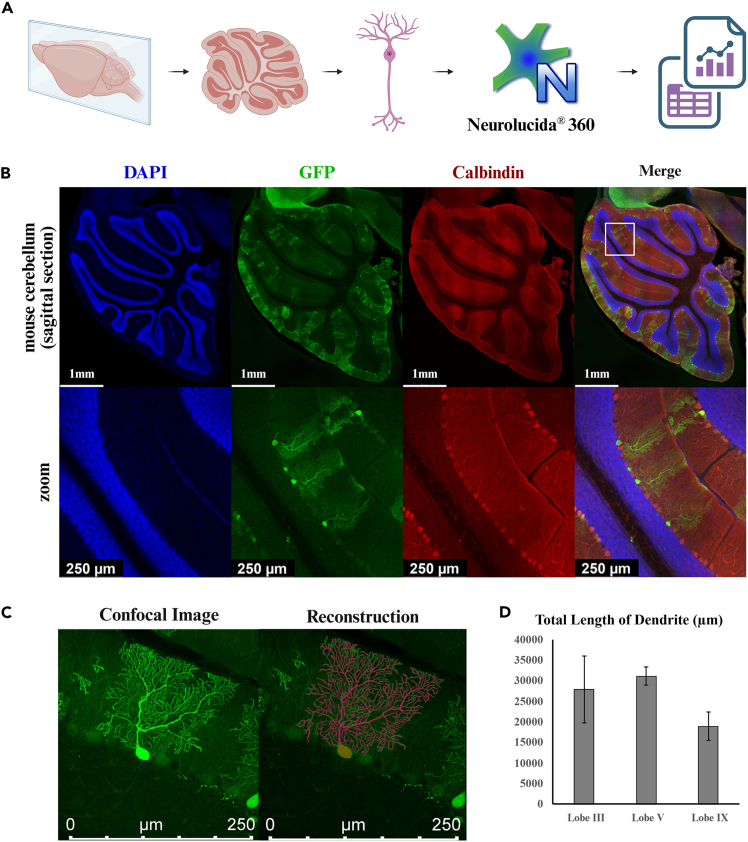
Representative images and results from the procedures (A) Individual Purkinje cells were resolved from sagittal sections of whole mouse cerebella for subsequent analysis with Neurolucida360. (B) Representative images of whole mouse cerebella (top) and zoomed in regions (bottom) are shown. The zoomed in region is denoted by the white box in the whole cerebellum merge. Sections were stained for DAPI, GFP, and calbindin. (C) The corresponding Neurolucida reconstruction for a representative wild type Purkinje cell is shown. (D) Total dendritic length is shown across 3 different cerebellar lobes. Error bars represent standard deviation, n = 3 cells, one cell per lobe analyzed from each mouse.

36. Image slides using an appropriate objective on a confocal microscope (we used 20×) with 0.1 μm z-step (see troubleshooting for guidance on this step, Figure 2E).
37. Export as TIFF files (images shown in Figure 3B, see troubleshooting for guidance on this step).

### Image reconstruction, export, and analysis

**Timing: Variable**

In this step we describe the handling of the microscopy images obtained from the previous step

38. Load TIF image stacks into MBF Bioscience’s Neurolucida 360 software using the “open image stack” option.
  a. Open the 3D window by clicking the 3D icon in the ribbon.
  b. Select the soma option and click the soma of your cell.
    ***Note:*** Holding control and using the click-wheel will expand and condense the cross hairs to call your soma.
  c. Select the dendrite option on the menu to your right. Manually reconstruct the tracings of dendritic structure (Figure 3C).
    ***Note:*** We found MBF Bioscience’s customer support to be a strong troubleshooting resource.
39. When complete, select export to Neurolucida Explorer on the top ribbon. This should open a new window.
  a. Select the combination of analysis you wish to run by using the “Analyze” tab on the top ribbon.

***Note:*** Here, we display the total length of dendrite (μm) for lobes III, V, and IX (Figure 3D).

## Expected outcomes

An overview of the sectioning, imaging, and analysis pipeline is given in Figure 3A. We used anti-GFP antibodies, and additionally stained with calbindin, a known Purkinje cell marker (Figure 3B). 1.65 × 10^10^ genome copies were injected in each hemisphere and we observed approximately 20% of the Purkinje cells were sparsely labeled throughout the Purkinje layer of the cerebellum. Purkinje cells in the superficial layer of the cortex seem to be transduced more efficiently when compared to Purkinje cells deeper in the cerebellar fissures. Investigators should use our reported viral titer and images (Figure 3B) as a starting point for their own optimizations. Each mounted slide contained 4–8 sagittal sections, and this labeling pattern was consistent across all sections.

Using Neurolucida 360, this degree of labeling was appropriate to reconstruct single Purkinje cells in three dimensions, easily distinguishing the dendrites from one plane to another (Figure 3C). However, GFP-labeled cells adjacent to one another and on the same z-plane were too interwoven to reconstruct. Purkinje cells were selected as candidates based on labeling signal, location in the lobe, and placement along the vermis of the cerebellum. For this protocol, we traced the dendrite of Purkinje cells manually from lobes III, V and IX and ran the “Neuron Summary” analysis in Neurolucida explorer, quantifying the total length of the dendrites (Figure 3D). Additionally, Neurolucida Explorer provides a range of analyses that can be run including convex hull^2^ and Sholl^3^ allowing for characterization of dendritic arbor and complexity.

## Limitations

The use of a thin glass needle for lateral ventricle injection is procedurally analogous to stereotaxic intracerebral injection, which is extensively characterized in the literature. Such procedures are well established to produce only localized tissue effects confined to the immediate vicinity of the needle tract. Sham injections performed under sterile conditions do not result in histopathologic changes beyond the tip of needle advancement, and no inflammatory markers have been detected surrounding the needle tract, confirming that a sterile invasive procedure of this type does not result in significant local inflammatory response.^4^ Similarly, the absence of significant microglial activation in stereotaxically injected brains has been confirmed by immunostaining when sterile surgical conditions are maintained.^5^ Taken together, the procedure described here is ideal for sparse Purkinje cell labeling; additionally, it is reasonable to assume the morphological characterizations assessed using this protocol are absent of confounding effects such as immunological activation or physical perturbation. Consistent with this, in our hands, injected animals are behaviorally indistinguishable from uninjected controls. However, there are four main limitations when adapting this protocol to suit other experimental questions. First, this labeling procedure relies heavily on AAV tropism, which has been defined for many, but not all, cell populations.^6^ Secondly, neonatal microinjections must be performed before postnatal day 3, after which the skull ossifies.^7^ Third, while we describe the viral titer that was effective in our studies, other neuronal populations are not likely to behave identically to cerebellar Purkinje cells and will require optimization. Lastly, only small amounts of injectant can be used on neonates, putting significant constraints on deliverable concentration.^8^

## Troubleshooting

### Problem 1

Surface tension and electrostatic interactions can make pipetting the viral mixture onto the parafilm difficult (step 7).

### Potential solution

Use low-retension pipette tips.

### Problem 2

Viral mixture is not being drawn into the borosilicate needle (step 10).

### Potential solution

Capillary action is sometimes slow. If the liquid is not immediately taken up, repeatedly remove and reinsert the needle tip into the 1 μL aliquot until it is entirely loaded into the needle. Take care not to pierce the parafilm with the needle.

### Problem 3

Air is injected during the microinjection procedure (step 12).

### Potential solution

This will result in a high chance of immediate death. During pup recovery, monitor for abnormal behavior or hydrocephaly. These events should become very rare after comfortability with the procedure is achieved (>98% survival). Defer to your institution’s guidelines on vertebrate animal handling.

### Problem 4

Maternal cannibalization occurs after neonatal injection (step 14).

### Potential solution

Rub urine-soaked cage bedding on gloves and recovery paper towel before cryoanesthesia. Change gloves and recovery towel between each litter to keep the odors from mixing.

### Problem 5

Incomplete or inadequate perfusion denoted by inflation of the lungs and intestines, fluid leaking from the eyes or nose, and/or blood retention in the circulatory system and liver (step 23).

### Potential solution

• The butterfly needle position was imprecise, or the pressure applied on the syringe was too great. Utilize less pressure during the perfusion procedure.
• If the perfusion becomes unproductive you can refill the syringe with 50 mL of 4% PFA solution and do a second perfusion. Reinsertion of the needle into the heart is generally not recommended, however it may be helpful to do so in this situation.

### Problem 6

Diffuse or lack of immunofluorescent signal when imaging (step 36).

### Potential solution

• Extended or intense exposure to light can reduce GFP signal. After extracting the brain from the skull, store the tissue in the dark as much as possible.
• Antibodies can be sensitive to fixation time. Reduce post-fix incubation time, or decrease the temperature.
• Use a higher concentration of antibodies. For such cases we suggest incubating the tissue directly on the microscope slide using a hydrophobic pen to contain the antibody solution. This reduces the volume required. Incubate in a dark humidified chamber. A microscope slide box with a wet paper towel on the bottom works well.

### Problem 7

Damage to the tissue seen under the microscope (step 37).

### Potential solution

Damage to the tissue usually occurs during extraction of the brain from the skull. Use smaller tools and cut along cranial sutures. Additionally, damage to the tissue can occur after vibratome sectioning. Never directly touch the tissue, instead manipulate the sections using the agarose leaflets.

## Resource availability

### Lead contact

Information and requests for resources and reagents should be directed to Fedor V. Karginov (karginov@ucr.edu).

### Technical contact

Technical questions on executing this protocol should be directed to Jordan J. Lillibridge (lillibridge.jordan@gmail.com).

### Materials availability

This study did not generate new unique reagents.

### Data and code availability

The protocol includes representative images for illustration purposes. Quantitative datasets are not presented.

## Acknowledgments

We thank the Riccomagno lab for training and guidance and the University of California, Riverside vivarium staff. The graphical abstract and figures for this publication were created with BioRender.com. This work was supported by an 10.13039/100000002NIH grant 1R21NS118390 to F.V.K. and 10.13039/100000001NSF grant 2235566 to M.M.R.

## Author contributions

J.J.L. optimized the procedures, collected the data, and drafted the article. S.V. drafted the article and made all the figures, including the use of BioRender.com to generate the graphical abstract. M.M.R. conceived the idea for using a microelectrode holder during his studies and edited the article. F.V.K. edited the article and served as the scientific advisor.

## Declaration of interests

The authors declare no competing interests.
